# Wide variation in susceptibility of transmitted/founder HIV-1 subtype C Isolates to protease inhibitors and association with *in vitro* replication efficiency

**DOI:** 10.1038/srep38153

**Published:** 2016-11-30

**Authors:** Katherine A. Sutherland, Dami A. Collier, Daniel T. Claiborne, Jessica L. Prince, Martin J. Deymier, Richard A. Goldstein, Eric Hunter, Ravindra K. Gupta

**Affiliations:** 1 Division of Infection and Immunity, UCL, London, UK; 2 Emory Vaccine Centre, Atlanta GA, USA

## Abstract

The *gag* gene is highly polymorphic across HIV-1 subtypes and contributes to susceptibility to protease inhibitors (PI), a critical class of antiretrovirals that will be used in up to 2 million individuals as second-line therapy in sub Saharan Africa by 2020. Given subtype C represents around half of all HIV-1 infections globally, we examined PI susceptibility in subtype C viruses from treatment-naïve individuals. PI susceptibility was measured in a single round infection assay of full-length, replication competent MJ4/*gag* chimeric viruses, encoding the *gag* gene and 142 nucleotides of *pro* derived from viruses in 20 patients in the Zambia-Emory HIV Research Project acute infection cohort. Ten-fold variation in susceptibility to PIs atazanavir and lopinavir was observed across 20 viruses, with EC_50_s ranging 0.71–6.95 nM for atazanvir and 0.64–8.54 nM for lopinavir. Ten amino acid residues in Gag correlated with lopinavir EC_50_ (p < 0.01), of which 380 K and 389I showed modest impacts on *in vitro* drug susceptibility. Finally a significant relationship between drug susceptibility and replication capacity was observed for atazanavir and lopinavir but not darunavir. Our findings demonstrate large variation in susceptibility of PI-naïve subtype C viruses that appears to correlate with replication efficiency and could impact clinical outcomes.

The successful global roll-out of antiretroviral therapy has resulted in approximately 15.8 million HIV positive individuals receiving antiretroviral therapy to date^1^. In resource-limited settings, 15–35% of patients experience therapy failure on their first-line treatment regimen (usually comprising 2 nucleoside reverse transcriptase inhibitors, NRTIs, and 1 non-nucleoside reverse transcriptase inhibitor, NNRTI) in the first two years^2^, frequently with high-level drug resistance to all components of the regimen^3^,^4^,^5^. WHO recommended second-line regimens include ritonavir boosted protease inhibitors (bPIs), particularly the PI lopinavir (LPV), and scale up of second line is underway^6^. Boosted PIs have also been used extensively in resource-rich settings as part of first-line regimens and have similar efficacy to NNRTI based regimens^7^,^8^.

Despite their widespread use, the viral genetic correlates of PI resistance are not fully understood. Treatment failure on PI-containing regimens frequently occurs in the absence of major resistance mutations, with less than 20% of patients developing major mutations in Protease^7^,^9^. Gag, a substrate of Protease, also affects PI susceptibility and contributes to PI resistance^10^. However, most previous mutations linked with PI resistance were observed in subtype B viruses and, in non-B subtypes, these mutations can be present as consensus or polymorphisms^11^,^12^,^13^,^14^. Additionally, our previous data using patient derived Gag-Protease sequences demonstrated that West African HIV-1 subtype CRF02_AG viruses displayed intrinsic reduced susceptibility to PIs and that their susceptibility to PIs pre-treatment was associated with treatment outcome^15^.

Subtype C HIV-1 is responsible for approximately 50% of infections globally and is most prevalent in Sub-Saharan Africa. PIs may target subtype C protease less efficiently and patients infected with subtype C viruses have poorer treatment outcomes on PI-based therapy^16^,^17^. Inclusion of co-evolved Gag affected LPV susceptibility of subtype C molecular clones^18^ and susceptibility of resistant protease from paediatric patients failing PI-therapy in *in vitro* phenotypic assays^13^. However, to date there are no data on the *in vitro* PI susceptibility of newly transmitted subtype C clinical isolates from untreated adults as measured using full-length replication competent chimeric viruses differing only in their patient-derived *gag-protease* genes^19^. We sought to study PI susceptibility and *in vitro* replication efficiency in a unique panel of subtype C chimeric viruses generated from acutely infected individuals enrolled in the Zambia-Emory HIV Research Project (ZEHRP) transmission cohort.

## Materials and Methods

### Study details

All participants were part of the ZEHRP discordant couples cohort, with subsequent HIV-1 transmission^20^,^21^. Subjects for this study were acutely infected recipients who seroconverted during the observation period^21^. Informed consent was obtained from all subjects and ethical approval for experimental protocols was obtained from both the University of Zambia Research Ethics Committee and the Emory University Institutional Review Board. All methods were carried out in accordance with guidelines and regulations of both the University of Zambia and Emory University.

Patient and clinical characteristics are shown for the twenty patients in Table 1. A positive correlation between patient RC and plasma viral load was previously reported in this patient cohort^21^. In addition an inverse correlation with CD4 was noted in that study, consistent with the notion of fitter viruses leading to more rapid disease progression.

### Plasmid construction

Patient *gag* and partial *protease* from the earliest seroconversion plasma sample was amplified and cloned into a subtype C infectious molecular clone MJ4, as previously described^21^. The resulting MJ4/*gag* chimeric vectors encoded full-length patient Gag, extending 142 nucleotides into Protease, corresponding to amino acid 40. For measurement of the contribution of particular mutations, Gag-Protease was cloned into Gag-Pol expression vector p8.9NSX+ as previously described^22^. Site directed mutagenesis was performed using QuikChange Lightening Site-Directed Mutagenesis Kit (Agilent) as per manufacturer’s instructions.

### PI susceptibility and replication capacity measurement

The replication capacity (RC) of 149 MJ4/*gag* chimeric viruses had been measured in a multi-round T cell replication assay, scored and categorised as previously described, as listed in Table 1^21^. Briefly, viruses were generated by transfection of 293 T cells and GXR25 cells were infected at a constant multiplicity. Viral production was measured in the supernatant and RC scores calculated, then viruses were categorised as ‘high’, ‘middle’ or ‘low’, based on the division of the distribution of viral RC scores into terciles^21^,^23^. For this study, ten viruses from each of the high and low RC categories were randomly selected. PI susceptibility of these twenty MJ4/Gag chimeric full-length, replication competent molecular clones was measured in a single round of infection as described previously^24^, in an assay adapted for replication competent virus, without a spinoculation step^25^. Susceptibility to the PIs lopinavir (LPV), darunavir (DRV) and atazanavir (ATV) was measured (NIH AIDS Reagent Program) and EC_50_ and EC_90_ were calculated for each PI. The PI susceptibility of the mutant viruses, generated in order to examine the effect of specific mutations in *gag* and *protease*, was measured in a single cycle phenotypic assay with VSV-g pseudotyped viruses produced using a triple-vector system, as previously described^24^.

### Analysis of genetic correlates

Patient *gag* and partial *pro* sequences were aligned at codon level using the ClustalW algorithm in MEGA 6.0^26^. Gag sequences were examined for the presence of mutations previously reported to contribute to PI resistance^13^. Protease sequences were analysed using the Stanford Drug Resistance Algorithm for the presence of known resistance mutations and polymorphisms^27^. Additionally, a mutual information statistical approach (with correction for multiple comparisons) was used to identify novel mutations in Gag associated with LPV EC_50_^28^.

## Results

### Large variation in PI susceptibility of viruses derived from PI-naïve patients

**Figure 1:**
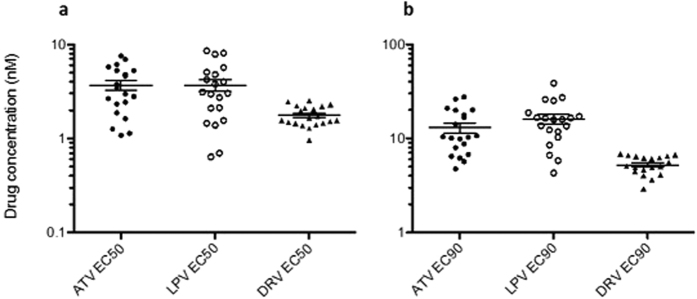
Variation in PI susceptibility of patient-derived viruses to PIs LPV and ATV. The EC_50_ (**a**) and EC_90_ (**b**) of twenty MJ4/gag chimeric full-length molecular clones derived from acutely infected patients infected with subtype C virus was determined using a single replication cycle assay to the PIs atazanavir (ATV), lopinavir (LPV) and darunavir (DRV). Data are representative of at least 2 independent experiments.

Up to 14-fold variation in susceptibility to the PIs ATV and LPV was observed across the 20 MJ4/Gag chimeric clinical virus isolates, with EC_50_ ranging 0.71–6.95 nM for ATV and 0.64–8.54 nM for LPV (Fig. 1a). Less variation in susceptibility to DRV was observed (0.96–2.55 nM). In addition, a similar distribution of PI susceptibility was observed when EC_90_ was measured (Fig. 1b).

### Identification of genetic correlates of PI susceptibility

Gag cleavage sites were examined for the presence of amino acid residues that might correlate with PI susceptibility based on previous literature^10^,^13^ (Supplementary Table S1). The MA/CA, CA/p2 and NC/p1 cleavage sites were largely conserved between the patient viruses and in comparison with consensus M sequence (Supplementary Table S1). Mutations previously implicated in PI susceptibility were present: 375 S (n = 2), 375 N (n = 17), I376V (n = 3), R380K (n = 7), G381S (n = 16), K436R (n = 2), S451N (n = 10) and P453L (n = 4) and P453T (n = 1). Of these, mutation R380K in the p2/NC site was more frequent in viruses with higher LPV EC_50_s and mutations P453L/T and I376V were enriched in the more susceptible viruses.

Additionally, the following known Gag non-cleavage site mutations previously associated with PI susceptibility^13^ were present in this cohort: K30R (n = 3), G62R (n = 2), R76K (n = 6), Y79F (9), T81A (n = 1), M200I (n = 1), H219Q (n = 5), I389T (n = 1) and T456S (n = 2) (Supplementary Table S2). Of these, only Y79F associated with more susceptible viruses and none appeared to correlate with reduced PI susceptibility.

As MJ4/Gag chimeric vectors encoded patient Protease up to residue 40, partial Protease was examined for resistance mutations, as defined by the Stanford Resistance Algorithm (http://hivdb.stanford.edu). Neither major nor minor resistance mutations in protease were identified, but polymorphisms were present at 9 Protease positions including I15V (n = 17), K20R (n = 2), E35D (n = 5) and M36I (n = 17) (Supplementary Table S3). Only E35D was enriched in viruses with reduced LPV susceptibility (median EC_50_ = 7.88 nM).

To enable the identification of novel mutations associated with PI susceptibility we employed a mutual information statistical approach. This approach yielded 10 amino acid positions statistically associated with LPV EC_50_: Gag 11, 18, 109, 119, 138, 182, 376, 380, 389 and 478 (Table 2). Three mutations clearly correlated with resistance to LPV: R18K, R380K and 478 L, and three with sensitivity: I376V, I389S/T/V and 478Q.

### Modest impact of mutations at Gag positions 390 and 389 on PI susceptibility

Having identified mutations correlated with PI susceptibility using an *in silico* approach, we aimed to quantify the relative contributions of key mutations in the variation in PI susceptibility observed by means of *in vitro* drug susceptibility testing. Two representative patient viruses were selected for further study – 170 (termed ‘resistant’ for this analysis) and 177 (termed ‘sensitive’). Site directed mutagenesis was used to revert the mutations correlating with reduced susceptibility in patient 170 Gag-Protease, and to introduce the corresponding ‘resistance’ mutations into patient 177 Gag-Protease. Mutants were assayed for PI susceptibility in a single cycle assay system. The effect of four amino acid residues significantly associated with susceptibility was studied: Gag 119, 380, 389 and protease 35, with mutants being assayed for PI susceptibility in a single cycle assay system.

**Figure 2:**
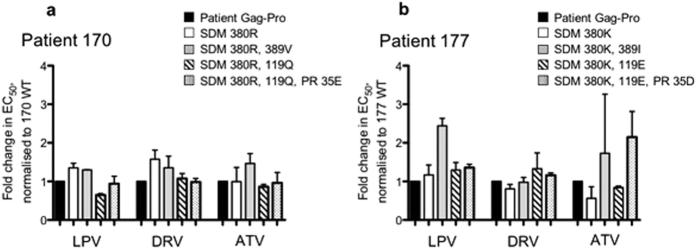
Direct contribution of identified mutations in Gag and Protease on PI susceptibility. PI susceptibility of (**a**) 170 (‘resistant’) and (**b**) 177 (‘sensitive’) and mutant viral vectors, in a PI susceptibility assay using VSV-g pseudotyped virus. PI susceptibility is expressed as a fold change in EC50 in comparison with the reference virus (black bars) which in panel (a) is 170 and in panel (b) is 177. Data are representative of at least 2 independent experiments.

Phenotypic PI susceptibility measurements did not demonstrate a direct effect of the reversion of resistance associated mutations to Gag 119Q, 380 R, 389 V and Protease 35E in Gag-Protease of patient 170, the ‘resistant’ virus. The reciprocal experiment introducing mutations Gag 119E, 380 K, 389I and protease 35D into patient 177 Gag-Protease demonstrated a small increase in EC_50_ (2.5 fold) for the mutations 380 K and 389I in combination, but other mutations showed no direct phenotypic effect when introduced singly or in combination (Fig. 2).

Finally, we hypothesised that there may be a correlation between PI susceptibility and HLA type in these patients, given that selective pressure on Gag can lead to CTL escape mutations in regions known to contribute to PI susceptibility^29^. We therefore examined HLA Class I haplotypes for participants and partners (Supplementary Tables S4 and S5) and determined associations with drug susceptibility. Due to the diversity in HLA types present and the relatively small number of patients, this analysis was inconclusive.

### Replicative capacity is associated with PI susceptibility

**Figure 3:**
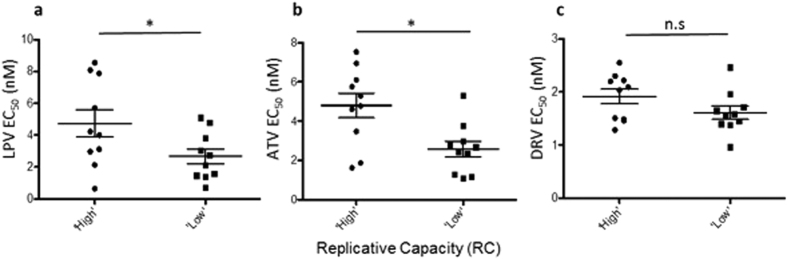
Correlation between PI susceptibility and viral replicative capacity. The EC_50_ of twenty MJ4/gag chimeric full-length molecular clones derived from acutely infected patients infected with subtype C virus was determined using a single replication cycle assay to the PIs atazanavir (ATV), lopinavir (LPV) and darunavir (DRV). Replicative capacity was determined by viral quantification of the supernatant of infected cells over multiple rounds of infection and then scored by terciles. PI susceptibility as a function of viral replication capacity is shown for the PIs (**a**) LPV (two sample t-test, p = 0.0497), (**b**) ATV (p = 0.0081) and (**c**) DRV (p = 0.1166). Data are representative of at least 2 independent experiments.

High RC viruses displayed significantly lower PI susceptibilities than low RC viruses to LPV (LPV mean EC_50_ 4.73 vs 2.66 nM, p = 0.0497) and ATV (mean 4.80 vs 2.57 nM, p = 0.0081), Fig. 3a and b. This numerical difference in susceptibility of high and low RC viruses to LPV and ATV was also observed when EC_90_ was measured with a trend towards statistical significance (Supplementary Figure S1). Significant variation in DRV susceptibility by RC was not observed at either the EC_50_ or EC_90_ (Fig. 3c and Supplementary Figure S1).

Given our observation that high RC viruses had significantly higher EC_50_ to the PIs LPV and ATV than low RC viruses, we compared mutations identified as associating with susceptibility with those previously reported to affect RC in the ZEHRP patient cohort^21^. A single residue identified in our mutual information analysis was also identified in the previous study: 119 A was associated with increased RC but alanine was not present at this position in our subset of patients (Table 2). Positions 373, 374 and 451 located within the Gag cleavage sites were also identified in the previous analysis as being associated with RC^21^. Alanine (A) at position 373 was associated with reduced RC and glutamine (Q) with higher RC, but neither of these residues were present in our patient subset. Mutations 374 V and 451 N were present in our patient cohort, but neither correlated significantly with LPV EC_50_ (Supplementary Table S1).

Five positions located outside of the Gag cleavage sites previously linked to PI resistance were identified in the previously published analysis as correlating with viral RC: 12, 30, 62, 76 and 370^21^. Of these mutations E12K, 62 K, R76K and 370 A were not statistically significant after correction for multiple comparisons and did not correlate with LPV EC_50_ in our patient subset (Supplementary Table S2). Mutation M30R was associated with higher RC and appeared to be enriched in patients with reduced PI susceptibility, but 30 R was only present in three high RC patients in this cohort.

## Discussion

This is the first study to describe the variation in PI susceptibility of HIV-1 subtype C using patient specific Gag-Protease sequences from PI-naïve adults. Previous data has indicated that pre-treatment PI susceptibility correlates with treatment outcome in non-B subtypes and being infected with subtype C virus was recently associated with poorer PI treatment outcomes in Sweden^15^,^17^.

We found that PI susceptibility to LPV, the most widely used PI in second-line therapy worldwide, varied by 14-fold in these MJ4/Gag chimeric patient viruses. ATV, a better tolerated PI that is increasingly used in second-line treatment, demonstrated 10-fold variation. This variation in susceptibility correlated with several mutations in Gag, including R380K which has been previously described in PI-experienced patients infected with subtype B virus^30^. Interestingly the mutations P453L and I376V, which were previously observed following PI exposure in patients often alongside major resistance mutations in protease, in fact correlated with increased susceptibility in this cohort^10^. Here, we observed little variation in DRV susceptibility. These data are reassuring given as viruses with variable susceptibility to lopinavir or atazanavir, remain fully susceptible to darunavir, a PI which is currently available as salvage therapy after second line failure in some settings.

We identified mutations correlating with PI susceptibility using mutual information analysis, which has previously been applied to examine PI resistance patterns in Gag and Protease, demonstrating a network of connected mutations across Gag and Protease in response to PI^28^. We were unable to demonstrate a significant direct effect of up to three mutations on PI susceptibility in our assay system, which is not surprising given the extensive variation throughout the length of Gag between patients. It is highly likely that the variation in susceptibility observed here is in fact conferred by a combination of mutations spread throughout Gag and possibly Protease, which makes the identification of the precise combinations present in each patient virus challenging. Mechanistic explanations for the effect of non-cleavage site mutations on drug susceptibility are likely to be diverse, and could include altered intra-molecular bonding as suggested by Parry *et al*. for a triad of matrix mutations in helix 4^31^. Alternatively, interactions may occur between amino acids that are apparently not in proximity based on sequence, suggesting physical interaction during virion maturation^28^.

Although we had access to HLA Class I data for the participants and partners (Supplementary Tables S4 and S5), we were unable to find an association between Gag mutations affecting drug susceptibility and HLA haplotype.

Our observation of an association between decreased PI susceptibility and increased RC of treatment-naïve viruses was unexpected and to our knowledge has not previously been described. The observation is important given that RC was previously shown to be associated with viral load. It appears to contradict the view that resistant viruses are less fit, as a direct result of the reduced RC conferred by major resistance mutations^32^. However, it is important to remember that the viruses here do not contain major resistance mutations and are PI-naïve. Furthermore, it is highly unlikely that their transmitted partners received PI-containing therapy. We recently demonstrated a correlation between RC and sensitivity to interferon alpha that might be linked to the observations presented here^33^, and further mechanistic investigation is warranted. Of note, viral load did not always correlate with RC (Table 1), consistent with the role of host factors in control of HIV replication^34^.

An advantage of this study is that replication competent HIV was used with an HIV-1 envelope, as opposed to VSV-g pseudotyped viruses used for most previous work on PI susceptibility in clinical isolates. This is particularly important given recent data demonstrating that PIs can affect viral entry through an interaction between Gag and Envelope, an effect that is masked when a VSV-g envelope is used^35^. Whilst full-length Protease from the patient was not included in the chimeric vectors, this actually strengthens support for the hypothesis that Gag can affect PI susceptibility in a mechanism that is independent of compensation for resistance mutations in Protease. The majority of known PI resistance mutations that occur in protease are located after amino acid 40, and hence were not derived from the patient in this cohort. Future work should evaluate the clinical impact of variation in baseline PI susceptibility on the outcome of second-line PI based ART in resource limited settings, in particular with a view to identification of consistent genetic signatures that could be applied in a clinical diagnostic setting through cheap point-of-care genotypic resistance tests^36^. Given the continuing roll out of standardised ART regimens coupled with significant rates of virological failure after one year on LPV/r based second-line therapy in resource limited settings^37^, it is vitally important that we fully understand the determinants of PI efficacy in second-line regimens for non-B subtypes.

## Additional Information

**How to cite this article**: Sutherland, K. A. *et al*. Wide variation in susceptibility of transmitted/founder HIV-1 subtype C Isolates to protease inhibitors and association with *in vitro* replication efficiency. *Sci. Rep.* **6**, 38153; doi: 10.1038/srep38153 (2016).

**Publisher's note:** Springer Nature remains neutral with regard to jurisdictional claims in published maps and institutional affiliations.

## Supplementary Material

Supplementary Data

## Figures and Tables

**Table 1:** Participant information; NA – not available.

Patient ID	CD4 Count	Viral Load (copies/mL)	Replicative Capacity Score	Replicative Capacity Category
219	447	1742240	2.554	High
170	489	470712	2.836	High
259	377	654980	2.138	High
173	465	452000	2.507	High
186	533	128040	0.392	Low
255	919	732560	1.151	Low
84	399	43200	2.507	High
30	NA	200745	2.931	High
40	NA	141897	1.307	Low
159	746	2449372	2.758	High
195	878	206000	0.768	Low
167	774	16500	3.091	High
154	471	316	0.449	Low
174	612	15328	2.756	High
105	NA	17000	1.198	Low
82	682	782	1.002	Low
51	715	28396	1.104	Low
55	500	91720	0.353	Low
270	573	273000	0.355	Low
177	421	949132	2.634	High

**Table 2:** Amino acid changes in Gag associated with LPV EC_50_ by mutual information analysis.

Patient Number	RC	LPV EC_50_ (nM)	11	Gag Amino Acid (numbered according to HXB2)								
				18	109	119	138	182	376	380	389	478
219	High	8.54	E	R	N	E	L	Q	I	R	I	P
170	High	8.08	E	K	N	E	L	Q	I	K	I	P
259	High	7.88	G	R	N	E	L	T	I	R	I	L
173	High	5.71	G	K	N	E	L	Q	I	K	I	M
186	Low	5.08	G	R	N	E	L	Q	I	K	DEL	L
255	Low	4.77	E	R	N	G	L	Q	I	R	I	L
84	High	4.22	G	K	N	E	F	Q	I	K	I	V
30	High	4.00	E	R	N	E	L	Q	I	K	I	S
40	Low	3.81	G	K	N	E	L	Q	I	R	I	L
159	High	3.11	E	K	N	K	L	Q	I	K	I	P
195	Low	3.02	E	K	N	E	L	Q	I	K	I	L
167	High	2.97	G	K	S	Q	H	S	V	R	S	P
154	Low	2.73	G	K	K	E	I	Q	I	R	I	P
174	High	2.13	G	K	K	K	L	Q	V	R	I	Q
105	Low	2.10	G	K	N	K	L	Q	I	R	I	Q
82	Low	1.56	D	A	N	E	L	Q	V	R	I	P
51	Low	1.45	G	K	N	K	L	S	I	R	T	DEL
55	Low	1.39	G	K	N	Q	H	S	I	R	I	Q
270	Low	0.70	E	K	K	K	L	Q	I	R	V	P
177	High	0.64	G	K	N	Q	I	Q	I	R	V	S
		**P value**	**0.00968**	**0.00389**	**0.00268**	**0.00145**	**0.00237**	**0.00691**	**0.00741**	**0.00039**	**0.00257**	**0.00301**
