# Supplementary material for: Wide variation in susceptibility of transmitted/founder HIV-1 subtype C Isolates to protease inhibitors and association with in vitro replication efficiency
Source: Sci Rep. 2016 Nov 30;6:38153. doi: 10.1038/srep38153 (PMC5128871; doi:10.1038/srep38153)
Supplement: Supplementary Data [file srep38153-s1.docx]

**Wide variation in susceptibility of transmitted/founder HIV-1 subtype C Isolates to protease inhibitors and association with *in vitro* replication efficiency**

Katherine A Sutherland^1^, Dami A. Collier^1^, Daniel T Claiborne^2^, Jessica L Prince^2^, Martin J Deymier^2^, Richard A Goldstein^1^, Eric Hunter^2^, Ravindra K Gupta^1^ *.

**Supplementary Table S1. Variation in Gag cleavage sites amongst patient-derived viruses**. The sequence at each of the Gag cleavage sites is shown for the 20 patients phenotyped. Viruses are ranked by LPV EC50 from high (8.5 nM for virus 219) at the top of the table to 0.6 nM at the bottom. Sequences are compared with the consensus M sequence using HXB2 numbering. Deletions are shown by an X.

| Patient Number | RC | Gag Cleavage Sites | | | | |
| --- | --- | --- | --- | --- | --- | --- |
|  |  | **MA/CA**  128-137 | **CA/p2**  359-368 | **P2/NC**  373-382 | **NC/p1**  428-437 | **p1/p6**  444-453 |
|  |  | VSQNY/PIVQN | KARVL/AEAMS | SATIM/MQRGN | ERQAN/FLGKI | RPGNF/LQSRP |
| 219 | High | -----/----- | -----/----- | NAN-M/I--S- | -----/----- | -----/--N-- |
| 170 | High | -----/----- | -----/----- | NTN--/--KS- | -----/---R- | -----/--N-- |
| 259 | High | -----/----- | -----/----- | S-S-L/----- | -----/----- | -----/----- |
| 173 | High | I----/----- | -----/----- | N-N--/--KS- | -----/----- | -----/--N-- |
| 186 | Low | -----/----- | -----/----- | STN--/--KS- | -----/----- | -----/----- |
| 255 | Low | -----/----- | -----/----- | NVN--/---S- | -----/----- | -----/----- |
| 84 | High | -----/----- | -----/----- | HGN--/--KS- | -----/----- | -----/--N-- |
| 30 | High | -----/----- | -----/----- | NTA--/--KS- | -----/----- | -----/--N-- |
| 40 | Low | -----/----- | --K--/----- | S-N-L/---S- | -----/----- | -----/----- |
| 159 | High | -----/----- | -----/----- | N-N--/--K-- | -----/----- | -----/H---L |
| 195 | Low | -----/----- | -----/----- | SIN--L--KS- | -----/----- | -----/--N-T |
| 167 | High | -----/----- | -----/----- | NVNV-/---S- | -----/----- | -----/A---L |
| 154 | Low | -----/----- | -----/----- | SXN-L/---S- | -----/---R- | -----/----- |
| 174 | High | -----/----- | -----/----- | T-NVL/---S- | -----/----- | -----/--N-Q |
| 105 | Low | -----/----- | -----/----- | N-S--/--—NT | -----/----- | -----/--N-- |
| 82 | Low | -----/----- | -----/----- | N-NV-/I--S- | -----/----- | -----/--N-- |
| 51 | Low | I----/----- | -----/----- | STN--/---S- | -----/---RL | -----/--N-- |
| 55 | Low | I----/----- | -----/----- | SXN--/---S- | -----/----- | -----/P---L |
| 270 | Low | -----/----- | -----/----- | KGN--/---S- | G----/----- | -----/--N-- |
| 177 | High | -----/----- | -----/----- | NTN--/---N- | G----/----- | -----/----- |

| **Pt** | **RC** | **LPV EC_50_** | **E12K** | **K30R** | **G62R** | **L75R** | **R76K** | **Y79F** | **T81A** | **K112E** | **M200I** | **H219Q** | **Q369H** | **V370** | **I389T** | **V390D/A** | **I401T/V** | **T456S** | **E468K** |
| --- | --- | --- | --- | --- | --- | --- | --- | --- | --- | --- | --- | --- | --- | --- | --- | --- | --- | --- | --- |
| MJ4 | - | - | N | M | Q | L | R | F | T | Q | M | Q | Q | A | I | I | I | T | E |
| 219 | High | 8.54 | K | M | K | F | R | F | T | Q | M | H | Q | A | I | I | I | T | E |
| 170 | High | 8.08 | K | R | Q | L | R | Y | T | Q | M | H | Q | V | I | V | I | T | E |
| 259 | High | 7.88 | K | M | Q | L | R | Y | T | Q | M | H | Q | A | I | V | I | S | E |
| 173 | High | 5.71 | K | M | R | L | R | Y | T | Q | M | H | Q | A | I | V | I | T | E |
| 186 | Low | 5.08 | K | M | K | L | K | Y | T | Q | M | H | Q | V | del | V | I | T | E |
| 255 | Low | 4.77 | K | M | K | V | K | Y | T | Q | M | H | Q | A | I | I | L | T | E |
| 84 | High | 4.22 | K | R | K | I | K | Y | T | Q | M | Q | Q | V | I | V | I | T | E |
| 30 | High | 4.00 | K | M | K | L | K | F | A | Q | M | H | Q | A | I | V | I | T | E |
| 40 | Low | 3.81 | K | M | K | L | T | Y | T | Q | M | H | Q | A | I | V | I | T | E |
| 159 | High | 3.11 | K | M | S | L | R | F | T | Q | M | Q | Q | A | I | V | I | T | E |
| 195 | Low | 3.02 | K | M | Q | L | R | F | T | Q | M | H | Q | A | I | V | I | T | E |
| 167 | High | 2.97 | K | R | K | L | R | Y | T | Q | M | Q | Q | A | S | V | I | T | E |
| 154 | Low | 2.73 | K | M | R | L | R | Y | T | Q | I | Q | Q | T | I | V | I | T | E |
| 174 | High | 2.13 | K | M | Q | L | R | F | T | T | M | H | Q | A | I | I | I | T | G |
| 105 | Low | 2.10 | K | M | K | L | R | F | T | Q | M | H | Q | A | I | V | I | T | E |
| 82 | Low | 1.56 | K | M | K | L | R | F | T | Q | M | H | Q | A | I | V | I | T | E |
| 51 | Low | 1.45 | K | M | K | L | Q | H | T | Q | M | H | Q | A | T | I | L | T | E |
| 55 | Low | 1.39 | K | M | K | L | K | F | T | Q | M | Q | Q | A | I | V | I | T | E |
| 270 | Low | 0.70 | K | M | Q | L | R | F | T | Q | M | H | Q | A | V | V | I | T | E |
| 177 | High | 0.64 | K | M | Q | I | K | Y | T | Q | M | H | Q | A | V | V | I | T | E |

**Supplementary Table S2. Variation in previously described non-cleavage site mutations (Giandhari et al. 2016).** The amino acid residue at each position previously described in relation to PI resistance, outside of the Gag cleavage sites, is shown for all twenty patient-derived sequences. Viruses are ranked by LPV EC50 from high (8.5 nM for virus 219) at the top of the table to low (0.6 nM) at the bottom.

| **Pt** | **RC** | **LPV EC_50_**  **(nM)** | **Protease polymorphisms (amino acid position and residue)** | | | | | | | | |
| --- | --- | --- | --- | --- | --- | --- | --- | --- | --- | --- | --- |
|  |  |  | **12** | **15** | **16** | **19** | **20** | **35** | **36** | **37** | **39** |
| MJ4 |  |  | S | V | G | I | K | E | M | S | S |
| 219 | High | 8.54 | T | V | G | V | K | D | I | D | P |
| 170 | High | 8.08 | S | V | G | I | K | D | I | N | P |
| 259 | High | 7.88 | T | V | G | I | K | D | I | N | P |
| 173 | High | 5.71 | S | V | G | I | K | D | I | N | P |
| 186 | Low | 5.08 | S | V | G | V | K | D | I | N | P |
| 255 | Low | 4.77 | S | V | E | I | K | E | M | N | P |
| 84 | High | 4.22 | T | V | G | I | K | E | I | N | P |
| 30 | High | 4.00 | S | I | G | T | R | E | I | N | P |
| 40 | Low | 3.81 | S | I | G | S | K | E | M | N | P |
| 159 | High | 3.11 | S | V | G | I | K | E | I | N | P |
| 195 | Low | 3.02 | S | V | G | T | R | E | I | N | P |
| 167 | High | 2.97 | S | V | G | I | K | E | I | N | P |
| 154 | Low | 2.73 | T | V | G | I | K | E | I | S | P |
| 174 | High | 2.13 | T | I | G | I | K | E | L | Q | P |
| 105 | Low | 2.10 | S | V | G | I | K | E | I | N | P |
| 82 | Low | 1.56 | S | V | E | I | K | E | I | N | P |
| 51 | Low | 1.45 | S | V | G | I | K | E | I | S | P |
| 55 | Low | 1.39 | S | V | G | I | K | E | I | N | P |
| 270 | Low | 0.70 | S | V | E | I | K | E | I | N | P |
| 177 | High | 0.64 | S | V | G | I | K | E | I | N | P |

**Supplementary Table S3. Variation in viral protease amongst patient-derived viruses.** The variable amino acid residues in protease are shown for all twenty patient-derived sequences. Viruses are ranked by LPV EC50 from high (8.5 nM for virus 219) at the top of the table to low (0.6 nM) at the bottom.

| Patient ID | Patient HLA type | | | | | |
| --- | --- | --- | --- | --- | --- | --- |
|  | **A1** | **A2** | **B1** | **B2** | **C1** | **C2** |
| 219 | A*290201 | A*330301 | B*3701 | B*4403 | C*0210 | C*0701 |
| 170 | A*2301 | A*3002 | B*1401 | B*4403 | C*0401 | C*0802 |
| 259 | A*3001 | A*3001 | B*4201 | B*5301 | C*0401 | C*1701 |
| 173 | A*0301 | A*6802 | B*1503 | B*4403 | C*0304 | C*0401 |
| 186 | A*020101 | A*2301 | B*1402 | B*4501 | C*0802 | C*1601 |
| 255 | A*0202 | A*2601 | B*070201 | B*5802 | C*0602 | C*0702 |
| 84 | A*300201 | A*3104 | B*4403 | B*5702 | C*0401 | C*1801 |
| 30 | A*3301 | A*3004 | B*350101 | B*5802 | C*0401 | C*0602 |
| 40 | A*290201 | A*6802 | B*070201 | B*5802 | C*0602 | C*0702 |
| 159 | A*3009 | A*6802 | B*0702 | B*4501 | C*0602 | C*0702 |
| 195 | A*010101 | A*2301 | B*570301 | B*8101 | C*1801 | C*1801 |
| 167 | A*3402 | A*3402 | B*4403 | B*4403 | C*0701 | C*0701 |
| 154 | A*3001 | A*3001 | B*4201 | B*4201 | C*0701 | C*0701 |
| 174 | A*020101 | A*3301 | B*4201 | B*4501 | C*1601 | C*1701 |
| 105 | A*2301 | A*3002 | B*4501 | B*570301 | C*0602 | C*18(01,02) |
| 82 | A*2301 | A*7401 | B*1401 | B*1503 | C*0210 | C*0802 |
| 51 | A*2301 | A*2301 | B*0801 | B*0801 | C*070101 | C*070101 |
| 55 | A*020101 | A*2902 | B*5301 | B*5801 | C*0401 | C*0701 |
| 270 | A*0201 | A*2902 | B*180101 | B*5802 | C*0602 | C*0704 |
| 177 | A*0201 | A*2301 | B*4901 | B*5801 | C*0302 | C*0701 |

**Supplementary Table S4: HLA Class I haplotypes amongst participants**

| Patient ID | Partner’s HLA type | | | | | |
| --- | --- | --- | --- | --- | --- | --- |
|  | **A1** | **A2** | **B1** | **B2** | **C1** | **C2** |
| 170 | A*2902 | A*6801 | B*3501 | B*4403 | C*0401 | C*0701 |
| 173 | A*3004 | A*7401 | B*350101 | B*530101 | C*0401 | C*0602 |
| 84 | A*0301 | A*2902 | B*4202 | B*5802 | C*0602 | C*1701 |
| 30 | A*3301 | A*3002 | B*0801 | B*1516 | C*0701 | C*1402 |
| 40 | A*3002 | A*680101 | B*4901 | B*5802 | C*0602 | C*0701 |
| 159 | A*0202 | A*0301 | B*1510 | B*5701 | C*0304 | C*0401 |
| 195 | A*3001 | A*3601 | B*1401 | B*1547 | C*0210 | C*0210 |
| 167 | A*0205 | A*2301 | B*1402 | B*580101 | C*0701 | C*0802 |
| 154 | A*0205 | A*3002 | B*570301 | B*580101 | C*0701 | C*1801 |
| 174 | A*3004 | A*6802 | B*1510 | B*5802 | C*0304 | C*0602 |
| 105 | A*3002 | A*3601 | B*0801 | B*5301 | C*0401 | C*0701 |
| 82 | A*0101 | A*3002 | B*4901 | B*5301 | C*0304 | C*0701 |
| 51 | A*2902 | A*3301 | B*1510 | B*4403 | C*0304 | C*0701 |
| 55 | A*3001 | A*3002 | B*4201 | B*5703 | C*1701 | C*1801 |
| 270 | A*0101 | A*3301 | B*530101 | B*5802 | C*0401 | C*0602 |
| 177 | A*7401 | A*8001 | B*1801 | B*4102 | C*0202 | C*1701 |

**Supplementary Table S5. HLA types of the transmitting partners for each patient.** Partner HLA type for patients 219, 259, 186 and 255 is removed because transmission to these patients occurred from an unrelated partner who was not enrolled in the cohort.

**Supplementary Figure S1: Reduced susceptibility (EC_90_) to PIs LPV and ATV for high RC viruses in comparison with low RC.**

P=0.0998

LPV EC_90_ (nM)

P=0.1454

P=0.6283

Replicative Capacity (RC)

ATV EC_90_ (nM)

DRV EC_90_ (nM)
